# Phenolic profile and antioxidant activity from peels and seeds of melon (*Cucumis melo* L. var. *reticulatus*) and their antiproliferative effect in cancer cells

**DOI:** 10.1590/1414-431X20176069

**Published:** 2018-03-01

**Authors:** P.M. Rolim, G.P. Fidelis, C.E.A. Padilha, E.S. Santos, H.A.O. Rocha, G.R. Macedo

**Affiliations:** 1 Departamento de Nutrição, Universidade Federal do Rio Grande do Norte, Natal, RN, Brasil; 2 Laboratório de Biotecnologia de Polímeros Naturais, Departamento de Bioquímica, Universidade Federal do Rio Grande do Norte, Natal, RN, Brasil; 3 Laboratório de Engenharia Bioquímica, Departamento de Engenharia Química, Universidade Federal do Rio Grande do Norte, Natal, RN, Brasil

**Keywords:** Melon residues, Antioxidant activity, Cancer cells, Free radical scavenging activity, Metal chelator, Cytotoxicity

## Abstract

Melon (*Cucumis melo* L.) has high economic value and in recent years, its production has increased; however, part of the fruit is wasted. Usually, inedible parts such as peel and seeds are discarded during processing and consumption. Extracts of melon residues were prepared and their phenolic compounds, antioxidants and antiproliferative activities were evaluated. Total phenolic compounds were found in hydroethanolic, hydromethanolic, and aqueous extracts, especially for melon peel (1.016 mg gallic acid equivalent/100 g). Flavonoids total content found for melon peel aqueous extract was 262 µg of catechin equivalent (CA)/100 g. In all extracts of melon peel significant amounts of gallic acid, catechin, and eugenol were found. For total antioxidant capacity, reported as ascorbic acid equivalent, the hydroethanolic and hydromethanolic extracts in peels and hydromethanolic in seeds were 89, 74, and 83 mg/g, respectively. Different extracts of melon showed iron and copper ions chelating activity at different concentrations, especially melon peel aqueous extract, reaching values of 61% for iron and 84% for copper. The hydroethanolic extract of melon peel presented a significant ability for hydroxyl radicals scavenging (68%). To assess the antiproliferative potential in human cancer cell lines, such as kidney carcinoma, colorectal carcinoma, cervical adenocarcinoma and cervical carcinoma, MTT assay was performed. The proliferation was inhibited by 20–85% at extracts concentrations of 0.1–1.0 mg/mL in all cancer cell lines. The results suggest that melon residues extracts display a high antioxidant activity in *in vitro* assays and have effective biological activity against the growth of human tumor cells.

## Introduction

Natural antioxidants, particularly present in fruits and vegetables, have had increased interest among consumers and the scientific community, due especially to nutritional and epidemiological evidence and the significant increase in the occurrence of chronic diseases, such as cancer. However, fruits and vegetables residues are often discarded, despite exhibiting excellent nutritional and functional characteristics (1).

Melon (*Cucumis melo* L.) is one of the most consumed and exported fresh fruits worldwide and its residue, peel and seeds, is commonly discarded. It has high economic value and is cultivated in several regions of the world due to its adaptation to different types of soil and climate. There is a high demand for production and commercialization of melon, and Brazil is one of the countries with great potential to supply this demand, with an area of approximately 17.5 thousand hectares used for melon cultivation (2).

Plant-derived compounds have been identified for prevention and treatment of cancer, such as resveratrol, lycopene and astaxanthin, and phenolic acids (3,4). Studies suggested that antioxidants from fruits and their residues can reduce the risk of cancer and related mortality, and consuming foods rich in polyphenols may lead to a lower incidence of cancers. Besides, the antioxidant potential and functional properties of nutrients from various natural sources have been investigated, mainly to replace the use of synthetic antioxidants in food products that can be a health hazard (5).

Investigation of *in vitro* antioxidant potential of plant extracts can be accomplished through a series of chemical and biochemical tests, promoting the discovery of new bioactive ingredients. These tests are important tools for screening synthetic and natural bioactive compounds as well as they can be employed in chemical, food and pharmaceutical industries (6).

Phenylpropanoid derivatives are an important group of low molecular weight phenolic acids, having hydroxycinnamic acid as the main example (ρ-coumaric, caffeic, ferulic and sinapic acids). They are usually linked to cell wall polysaccharides, mainly hemicellulose and lignin units (7). Flavonoids are part of a group of phenolic acids widely distributed in nature and their presence in vegetables may be related to defense functions and attraction of pollinators, and flavoring, bactericidal, fungicidal, astringent and anti-inflammatory effects (8). Tannins are also sources of phosphorus and antioxidants.

Different types of polyphenols (phenolic acids, hydrolysable tannins, and flavonoids) have also shown antiproliferative effects. Studies demonstrated a complex interaction between the generation of reactive oxygen species and cellular damage and carcinogenesis. The level of oxidative stress is commonly increased in pathological conditions such as inflammation, radiation, exposure to ultraviolet light, iron overload, and varied intake of carcinogenic chemical compounds. Thus, oxidative stress may be the base of the iceberg of carcinogenesis (9).

The use of antioxidants as preservatives in food products to extend their shelf life and maintain the quality, as natural color and antioxidants properties, is an effective alternative that is currently widely used, aiming at foods that are more healthy and functional.

## Material and Methods

### Cell culture and chemicals

Ethanol, methanol, sulfuric acid, potassium ferricyanide, ferrous sulfate (II), and aluminum chloride were obtained from Merck (Germany). Diphenylpicryl hydrazine (DPPH), catechin, and 3-(4,5-dimethylthiazol-2-yl)-2-5-diphenyltetrazolium bromide (MTT) were purchased from Sigma Chemical (USA). Sodium chloride was purchased from Sigma Chemical. HeLa cells (ATCC¯ CCL-2), SiHa cells ATCC¯ (HTB-35), 786-0 (ATCC¯ CRL-1932, HT-29 (ATCC¯ HTB-38), and Embryo fibroblast 3T3 (ATCC¯ CCL-164) were provided by Dr. Hugo Oliveira Rocha, Department of Biochemistry, UFRN, Brazil. Cell culture medium components (Dulbecco^'^s Modified Eagle Medium, DMEM) and trypsin were obtained from Cultilab (Brazil). Sodium bicarbonate, sodium pyruvate and phosphate buffered saline (PBS) were purchased from Invitrogen Corporation (Canada). All other solvents and chemicals were of analytical grade.

### Obtaining extracts

In order to analyze antioxidant capacity and anticancer properties different extracts were prepared from peel and seeds of melon (*Cucumis melo* L. *reticulatus* group). Initially, 3.0 g of each sample were weighed in Erlenmeyer flasks and 50 mL of distilled water was added, followed by agitation in a shaker at 130 rpm for 60 min at 22±2°C. The material was vacuum filtered using filter paper and submitted to a sequential extraction with water, hydro-methanolic solution (30:70 v/v) and hydro-ethanolic solution (30:70 v/v). The filtrates obtained were evaporated on a rotary evaporator and lyophilized.

### Total phenolic compounds analysis

The dosage of total phenolic acids was performed by the method of Folin-Ciocalteu (10). Readings were taken at 760 nm and total phenolic acids are reported in mg of gallic acid equivalents (GAE)/100 g of sample.

### Total flavonoid assay and total tannins

The flavonoid content was determined according to the method described by Saravanan and Parimelazhagan (11). A 0.5 mL extract aliquot (1 mg/mL) was mixed with 2 mL distilled water and subsequently with 0.15 mL of 5% NaNO_2_ solution. After 6 min, 0.15 mL of 10% AlCl_3_ was added and the mixture was allowed to stand for 6 min, and then 2 mL of 4% NaOH solution was added to the mixture. Distilled water was immediately added to bring the final volume to 5 mL, and then the mixture was thoroughly mixed and allowed to stand for another 15 min. Absorbance of the mixture was determined at 510 nm against the water blank. Catechin was used as the standard compound for the quantification of total flavonoids and the results were reported as catechin equivalents (CE).

The determination of tannins was done in the hydromethanolic extracts of melon peel and seeds and was performed using the vanillin method. In this assay both the leucoanthocyanidins (catechins) and the proanthocyanidins (condensed tannins) reacted with vanillin in the presence of HCl to produce a red condensed product, detected by spectrophotometer (12).

### Phytochemical analysis by thin layer chromatography (TLC)

Qualitative analysis was performed using the TLC with adsorbent aluminum and chromatography plates with silica gel 60. The mobile phase was: ethyl acetate (EtOAc), formic acid, water, and MeOH (9.4:0.5:0.8:0.4 v/v/v/v). After the chromatographic development, the TLC was analyzed under UV light at a wavelength of 254 nm. The TLC analysis was performed using as standard authentic samples of stigmast and β-sitosterin (Sigma-Aldrich) and vanillin sulfuric as universal processing.

### Determination of polyphenols by high performance liquid chromatography (HPLC)

The polyphenols in the extract as well as in the diluted phase were identified by HPLC using the chromatographic platform Accela (Thermo Scientific, USA); the system has a diode array detector (running at 220–360 nm) as well as an automatic sample injector and automatic fraction collector. For the experiments, a Shim-pack CLC-ODS(M) column with 250×4.6 mm (Shimadzu, Japan) was used. Elution was carried out in gradient under a flow rate of 1000 μL/min and oven temperature of 30°C, by the mixture of 1.0% acetic acid (v/v) (*A*) and acetonitrile (*B*) in the following proportions: 100% *A* as initial condition, 100 to 70% *A* at 10 min, 70 to 30% *A* at 15 min, 30 to 0% until 25 min. Finally, elution was maintained only with acetonitrile for a further 5 min. Samples were filtered using a 0.22 µm membrane and assayed in three replicates. The standards used were gallic acid, salicylic acid, catechin, ellagic acid, quercetin, vanillin, eugenol, and vanillic acid.

### *In vitro* antioxidant activity

The assay is based on the reduction of Mo (VI) to Mo (V) by melon residue extracts and subsequent formation of a green phosphate/Mo (V) complex at acidic pH levels. Tubes containing melon waste extracts and reagent solution (0.6 M sulfuric acid, 28 mM sodium phosphate, and 4 mM ammonium molybdate) were incubated at 95°C for 90 min. After the mixture had cooled to room temperature, the absorbance of each solution was measured at 695 nm. Total antioxidant capacity is reported as ascorbic acid equivalent (13).

### Hydroxyl radical scavenging activity

The scavenging activity of melon residue extracts against the hydroxyl radical was investigated using Fenton^'^s reaction (Fe_2_ + H_2_O_2_→Fe_3_+ OH− + OH˙). These results are reported as the inhibition rate. Hydroxyl radicals were generated using 3 mL sodium phosphate buffer (150 mM, pH 7.4), which contained 10 mM FeSO_4_·7H_2_O, 10 mM EDTA, 2 mM sodium salicylate, 30% H_2_O_2_ (200 mL) and varying extract concentrations. In the control, sodium phosphate buffer replaced H_2_O_2_. The solutions were incubated at 37°C for 1 h, and the presence of the hydroxyl radical was detected by monitoring absorbance at 510 nm. Gallic acid was used as the positive control (14).

### Ferrous Ion [Fe(II)] and copper chelating activity

The ferrous ion chelating ability of samples was investigated using melon residue extracts at different concentrations applied with the reaction mixture, which contained FeCl_2_ (0.05 mL, 2 mM) and ferrozine (0.2 mL, 5 mM). The mixture was shaken and incubated for 10 min at room temperature, and the absorbance of the mixture was measured at 562 nm against a blank. EDTA was used as the positive control (15–17).

### Reducing power

The reducing power of the samples was quantified as described previously. Briefly, 4 mL of reaction mixture, containing different sample concentrations in phosphate buffer (0.2 M, pH 6.6), was incubated with potassium ferricyanide (1% w/v) at 50°C for 20 min. The reaction was stopped by TCA solution (10% w/v). The solution was then mixed with distilled water and ferric chloride (0.1% w/v) solution and the absorbance was measured at 700 nm. The result is reported as a percentage of the activity shown by 0.2 mg/mL of vitamin C.

### Antiproliferative activity

The antiproliferative activity of extracts of melon peel and seeds was investigated with four tumor cell lines: HeLa and SiHa (human cervix tumor cells), 786–0 (kidney carcinoma), HT-29 (colon carcinoma), and normal cell 3T3 (mouse fibroblasts). The cell viability was evaluated by enzymatic reduction of 3-(4,5-dimethylthiazol-2-yl) -2,5 diphenyltetrazolium bromide (MTT) to formazan crystals, which reflects the functional state of the respiratory chain (18).

To determine the cytotoxicity by MTT assay, cells were seeded onto 96-well plates at a density of 5×10^3^ cells/well and allowed to attach overnight in 300 μL medium fetal bovine serum (FBS)-free incubated at 37°C, 5% CO_2_. The medium was then removed and 300 μL of medium and FBS was added, followed by melon extracts at a final concentration of 0.1, 0.25, 0.5, and 1.0 mg/mL. Cells were allowed to grow under these conditions for 24, 48, and 72 h at 37°C at 5% CO_2_. After incubation, traces of melon extracts were removed by washing the cells twice with 200 μL of PBS and applying 100 μL of fresh medium and 10 μL of MTT (5 mg/mL) dissolved in PBS to determine the effects of the melon residue extracts on cell proliferation. Cells were then incubated for 4 h at 37°C, 5% CO_2_. To solubilize the product of MTT cleavage, 100 μL of isopropanol containing 0.04 N HCl was added to each well and thoroughly mixed using a multichannel pipette.

Quantification of absorbance was performed in 96-well plate reader at a wave length of 570 nm using a Multiskan Ascent Microplate Reader (Thermo Labsystems, USA). The percent inhibition of cell proliferation was calculated as follows: % cell proliferation = Abs. 570 nm Control − Abs. 570 nm sample/Abs. 570 nm Control × 100. The inhibitory concentration (IC_50_) values were calculated from the plot of antiproliferative activity against the concentrations of the extracts.

### Statistical analysis

Results are reported as means±SD. Analysis of variance (ANOVA) and means testing were performed at a 95% confidence interval (P<0.05) using Statistica software version 7.0. The graphics were performed using Origin version 8.0. The Pearson product-moment correlation was also used to determine the relationship between antioxidant activity and total phenolic compounds. All experiments were repeated three times.

## Results and Discussion

### Phytochemical analysis, total phenols, total flavonoids, and tannins

**Figure 1.**
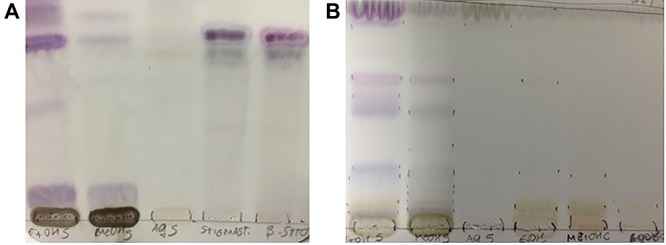
Thin layer chromatography of the melon seed (*A*) and peel (*B*) extracts with stigmasterol and β-sitosterol standards. AcOET: MetOH 5:5:0.5. AcOET: Formic acid: water: MetOH 9.4:0.5:0.8:0.4.

The phytochemical profile was performed using chemical reactions and identification analysis by thin layer chromatography. The phytochemical analysis provides relevant additional information and detects the presence of secondary metabolites in plants, so that the isolation of active principles can be achieved. An important parameter to be considered is the retention factor (Rf), which is the ratio of distance traveled by the substance of interest and the distance traveled by mobile phase (19). The hydromethanolic and hydroethanolic seed extracts had a diversity of compounds of about 6 bands. Bands in yellow were detected in ethanol and methanolic extracts of the peel, characteristic of saponins (Rf 0.76). The presence of brownish-stained bands suggesting the presence of phenolic compounds were also seen (Figure 1).

The observation of dark spots under UV light at 254 ηm indicates the presence of chromophore substances. As shown in Figure 1, it appears that all fractions showed blue-violet color bands, suggesting the presence of terpenes. Bands in purple, brown and yellow were observed, suggesting the presence of terpenes and saponins. Terpenes, also known as terpenoids, are a phenolic group of small structural and functional variety (20).

Phytochemicals have great physiological and morphological importance to plants, performing structural roles with protective functions against pathogens and predators to ensure reproduction through acquired resistance by the fruit. These molecules, including phenol compounds, are synthesized by the secondary metabolism maintenance and response mechanisms to offensive conditions such as ultraviolet radiation, absorption of heavy metals, temperature, and acidity, among others (21). In addition, evidence suggests that their use in food, especially in fruits and vegetables, provide antioxidant properties (22), and antithrombotic, anti-inflammatory, antiallergic, cardioprotective, antitumor (23), and antimicrobial actions (24).

The results for the evaluation of total phenolic compounds, total flavonoids and tannins are shown in Table 1. The solvents used for the extraction of phenolic compounds from melon residues were water, methanol and water (70:30 v/v), and ethanol and water (70:30 v/v). It is noteworthy that all solvents were effective in extracting the bioactive compound, highlighting the hydromethanol solution, which showed high solubility for the phenol. One sequential extraction was followed by extraction with water, followed by hydromethanol solution and finally hydroethanol, producing 1.106.88 mg of GAE/100 g of melon peel flour, and 256.57 mg GAE/100 g melon seed flour.

**Table 1.** Concentrations for the bioactive compounds of melon (*Cucumis melo* L. var. *reticulatus*) residue extracts.

Bioactive compound (μg/mL)	Melon seed extracts			Melon peel extracts		
	Aqueous	Ethanolic	Methanolic	Aqueous	Ethanolic	Methanolic
GA	10.80	0	0	8.83	96.35	100.01
SA	0.18	0.22	0.26	0.11	1.01	0.94
EA	0.07	0.06	0.06	0.02	0.19	0.15
CAT	1.77	0.90	1.07	4.10	26.18	27.44
QUER	0.10	0.16	0.15	0.08	0.25	0.37
VAN	0.70	0.93	1.14	0.39	0.17	0.37
EUG	0.18	0.35	0.31	0.37	1.58	1.55
VA	1.02	0.58	0.67	0	0	0

GA: gallic acid; SA: salicylic acid; EA: ellagic acid; CAT: catechin; QUER: quercetin; VAN: vanillin; EUG: eugenol; VA: vanillic acid.

For total phenolic compounds, the hydromethanolic peel extract showed a statistically higher content of the bioactive compound differing from all other extracts (P<0.05). The aqueous ethanolic extracts, although statistically different (P<0.05), extracted lesser amount of total phenols. In all extraction conditions (type of solvent, time, and temperature) the seed extracts showed a lower phenolic content than the peel extracts.

The solubility of these antioxidants in a specific solvent is a peculiar feature of phytochemicals in the food matrix, which explains the lack of a universal procedure for measuring antioxidant capacity and phenolic content. The mechanism for sequential extraction in food using different extractors is commonly used, as it provides higher bioactive affinity and improves bioavailability (25,26). The use of water in the solvent mixtures is important since it helps to break the complex solute matrix due to its high polarity, thus allowing a greater extraction of the solute from the substrate (27). The type of solvent and its polarity may affect the transfer of an electron and transfer of a hydrogen atom, which are key aspects in the measurement of antioxidant capacity (28).

In this study, total phenols content in melon residues were influenced by the type of solvent. In peel extracts, it ranged from 110.7 to 703.14 mg GAE/100 g of dry residue and in seed extracts, it ranged from 69.77 to 111.65 mg GAE/100 g of dry matter. The concentration of total phenols, following the sequential extraction in the peel extracts, was 4.3 times higher than the seed extracts. It is possible that pigments and tannins in the peel as well as the presence of total flavonoids contributed to the high polyphenol content.

Similar to phenols, Babbar et al. (29), who investigated the phenolic content in different types of fruit waste with methanolic extracts, reported values of 17.9 mg GAE/g for lychee seed, 24.6 mg GAE/g for lychee peel, 37.4 mg GAE/g for grape seed and 3.8 mg GAE/g for banana peel. Anagnostopoulou et al. (30), evaluating the content of bioactive compounds in orange peel in methanolic extract, concluded that total phenols value was 66.9 mg GAE/100 g of dry peel, a lower value than our findings.

The concentrations of carotenoids in melon peel and seed residues in this study were 96 and 9.59 µg/g, respectively. Chlorophyll and carotenoid levels possibly decreased after drying the residues, especially in the melon peel as it lost its green color after this processing. It is known that at room temperature, chlorophylls (a and b) are stable, but when temperatures rise above 50°C, chlorophyll levels can be affected. The decreased levels of chlorophyll following heat treatment are due to the conversion of chlorophyll to pheophytin, which is attributable to a change in pH during thermal processing (31).

In this study, residues in their fresh form and the flour obtained after drying were stored under freezing temperature in plastic containers but without vacuum, which probably contributed to the decrease in the carotenoid content. The exclusion of oxygen, light protection, as well as low temperatures decrease the decomposition of carotenoid during storage (32).

The results showed a high content of total phenols, total flavonoids, tannins, and carotenoids in the extracts, as the original matrix is a plant biomass based on macromolecules lignin, hemicellulose, and cellulose. The lignin is rich in polyphenols and aromatic groups, contributing significantly to the content of aromatic rings and hydroxyl radicals, with one notable phytochemical source of phenolic compounds.

### Determination of polyphenols by HPLC

**Figure 2.**
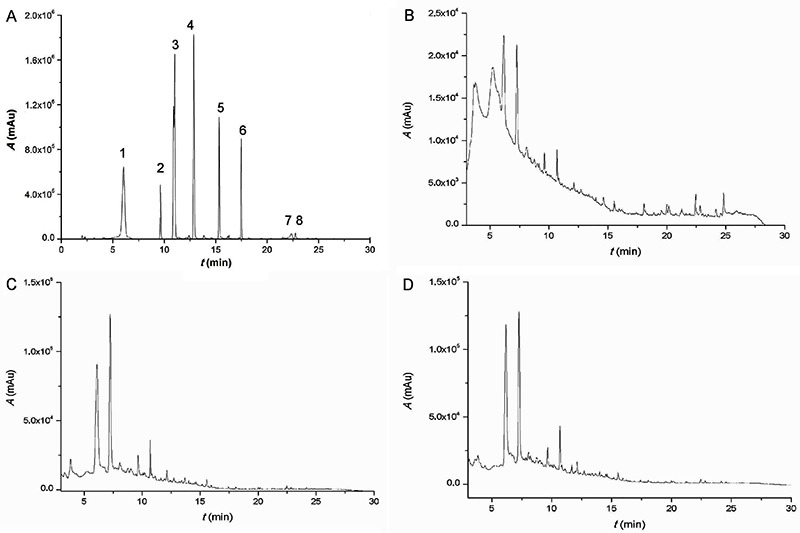
High performance liquid chromatography (HPLC) results of melon peel extracts. *A*, Standard calibration curve. *B*, Aqueous extract. *C*, Hydroethanolic extract. *D*, Hydromethanolic extract. Standards: 1: gallic acid; 2: salicylic acid; 3: ellagic acid; 4: catechin; 5: quercetin; 6: vanillin; 7: eugenol; 8: vanillic acid.

To support the analysis of antioxidant capacity and antiproliferative effects, we identified the bioactive compounds in the different melon residue extracts. According to Table 1, extracts had different values, which can be attributed to the difference in the total phenolic content as well as in the structure and exposure of phenols in the peel and seeds. The melon peel is more exposed to the environment stress conditions; this may have caused a higher phenolic content than in the seeds. In the peel extracts, higher levels of gallic acid, catechins, salicylic acid and eugenol were detected. In the melon seed extracts, the bioactive compounds identified were gallic acid, catechins, vanillic acid, eugenol and vanillin, however in lower concentration than the peel. (Table 1; Figure 2)

### Antioxidant activity

Analyses of antioxidant properties were performed using various types of chain reactions: initiation (total antioxidant capacity, (TAC), reducing power), propagation (metal chelating), termination (sequestration of hydroxyl radicals, superoxide, DPPH), and oxygen reactive antioxidant capacity (ORAC) test.

All extracts obtained from melon residues exhibited TAC activity reported as ascorbic acid equivalents (AAE) (Table 2). The presence of antioxidant activity in this assay indicates that extracts interacted with electron donating systems to minimize the attack of free radicals. The ethanol extract of melon peel flour showed the highest activity (88.71 mg/g AAE), followed by its methanol extract (74.48 mg/g AAE). No difference was found between ethanolic and methanolic peel extracts. No statistically significant difference was found for seed and peel methanolic extract in the TAC assay. However, a higher antioxidant capacity was found compared to other extracts, with 82.98 mg/g AAE. All aqueous extracts had low activity, reaching 34.71mg/g AAE for seeds and 40 mg/g AAE for peel (Table 1).

**Table 2.** Quantification of total phenolic compounds, total flavonoids and tannins and antioxidant properties (total antioxidant capacity, TAC), reducing power, DPPH radical-scavenging effect and ORAC assay of melon peel and seed extracts.

Bioactive compounds and antioxidant activities	Melon peel extracts (dry extract)			Melon seed extracts (dry extract)		
	Aqueous	Hydromethanolic (70:30 v/v)	Hydroethanolic (70:30 v/v)	Aqueous	Hydromethanolic (70:30 v/v)	Hydroethanolic (70:30 v/v)
Total phenols compounds (mg GAE/100 g)	110.7±15.03^c^	703.1±12.65^a^	203.1±7.17^b^	75.2±7.39^b^	111.7±12.36^a^	69.77±12.78^b^
Total flavonoids content (µg CE/100 g)	262±0.20^b^	125±0.10^a^	104±0.15^c^	109±0.40^a^	93±0.20^a^	99±0.30^a^
Tannins (µg CE/100 g)	nd	309±0.20^a^	nd	nd	146±0.30^b^	nd
TAC (mg AAE/g)	40.3±2.04^b^	74.5±2.76^a^	88.7±2.99^a^	34.5±1.98^b^	82.9±2.54^a^	49.8±1.76^b^
Reducing Power (%)	96.1±2.10^a^	17.5±1.35^b^	17.1±1.89^b^	25.3±1.79^b^	91.2±2.66^a^	91.6±2.78^a^
DPPH activity scavenging (%)	28.6±0.31^a^	30.1±0.23^a^	30.1±0.16^a^	1.09±0.54^c^	4.8±1.17^b^	6.2±0.43^a^
Total ORAC value (mmol TE/100 g)	12.2±0.67	17.4±1.02	19.49±0.97	2.75±0.18	20.2±0.66	21.9±1.87

Data are reported as means±SD with experiments performed in triplicate. GAE: gallic acid equivalent; CE: catechin equivalent; TE: trolox; nd: not determined. DPPH: 1,1-diphenyl-2-picrylhydrazyl assay for antiradical activity; ORAC: oxygen reactive antioxidant capacity. Antioxidant properties results were evaluated at 1.0 mg/mL concentration. TAC results are reported as ascorbic acid equivalent (AAE). Different letters indicate significant differences between different extracts of each residue at the same concentration (P<0.05, ANOVA).

The correlation tests showed high and positive coefficient indicating a strong correlation between these phytochemicals and antioxidant activity. The Pearson correlation coefficient for hydroethanolic extracts of peel and seeds were r=0.823 and r=0.881, respectively.

The peel aqueous extracts, and seed hydroethanolic and hydromethanolic extracts presented dose-dependent reducing ability above 90% at a concentration of 1.0 mg/mL (Table 2). It is known that a high reducing activity at low concentrations indicates antioxidant potential. One study reported that the reducing power of bioactive compounds was associated with antioxidant activity, since it is related to their ability to transfer electrons (33). The seed methanolic extract showed high total antioxidant capacity and high reducing activity suggesting that it can reduce or inhibit the initial phase of the oxidation process.

All extracts exhibited potential for chelating copper and iron metals, demonstrating their performance in the propagation phase. The different extracts of melon peel showed antioxidant activity by chelating iron ions in different concentrations (0.5 to 2.5 mg/mL) reaching 61% for the aqueous extract, followed by 49% and 46% for hydroethanolic and hydromethanolic extracts, respectively (Figure 2).

In the melon peel aqueous extract, eight compounds were identified: gallic acid, salicylic acid, ellagic acid, catechin, quercetin, vanillin, eugenol, and vanillic acid. The hydromethanolic and hydroethanolic peel extracts presented similar chemical profiles, with several compounds eluting with the same retention times. Chromatograms of peel extracts exhibited many signals, indicating the presence of varied bioactive compounds (Figure 2). The melon seed extracts, on the other hand, showed a chromatographic profile with few signs, indicating less diversity in bioactive compounds.

A study investigated the phytochemical composition and functional properties of melon peel (*maazoun*cultivar) and found that it is rich in antioxidants as polyphenols and flavonoids (332 and 95.46 mg/100 g extract, respectively). The obtained results indicate that hydroxybenzoic acids and flavones constitute their main phenolic classes. 3-hydroxybenzoic acid is the major phenolic compound in the melon peel with 33.45 mg/100 g, followed by apigenin-7-glycoside (29.34 mg/100 g) (34). The differences with our results are possibly due to climate and environmental variations of the cultivation region and to extraction methods. Nevertheless, all the extracts evaluated presented interesting antioxidant activities.

Metals such as copper (Cu), iron (Fe), selenium (Se), and zinc (Zn) are essential to living organisms in very small amounts. Excessive and chronic exposure leads to many detrimental effects on tissues and organs, eventually resulting in carcinogenesis. The molecular mechanisms by which metals act are not completely understood. It is known that metals have the potential to generate reactive oxygen species and thus alter the redox status of cells, which is considered the most important mechanism of metals-induced carcinogenesis. The production of reactive oxygen species induced by metals has been implicated in the initiation of cellular damage and the stimulation of inflammatory processes, which can lead to cancer development (35).

**Figure 3.**
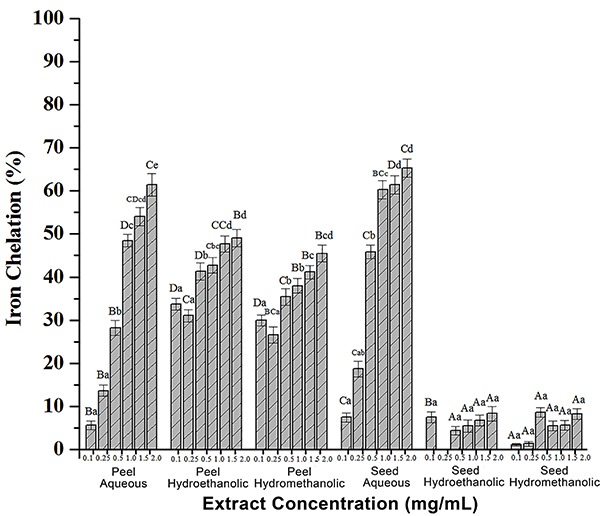
Antioxidant activity of melon peel and seed extracts by iron chelating effect. Different lower-case letters indicate a significant difference between melon residues extracts when compared at each concentration. Different upper-case letters indicate a significant difference between melon residues extracts at the same concentration (P<0.05, ANOVA). Data are reported as means±SD of 3 determinations.

The phenolic ability in *in vivo* metals chelation is still uncertain, but the use of phenols has been suggested for the treatment of diseases related to metal overload such as hemochromatosis (iron overload) and Wilson's disease (copper overload). We found that the aqueous extracts of melon peel presented iron ions chelation in 1.5 and 2.0 mg/mL concentrations, chelation ability of 54 and 61%, respectively. Seed aqueous extracts of 1.0, 1.5, and 2.0 mg/mL concentrations showed iron chelating activity of 60, 61, and 65%, respectively. The hydroethanolic and hydromethanolic peel extracts had a chelation capacity below 50%, and at higher concentrations, 49 and 45%. The hydromethanolic and hydroethanolic seed extracts showed no significant chelation activity, with values below 10% (Figure 3).

**Figure 4.**
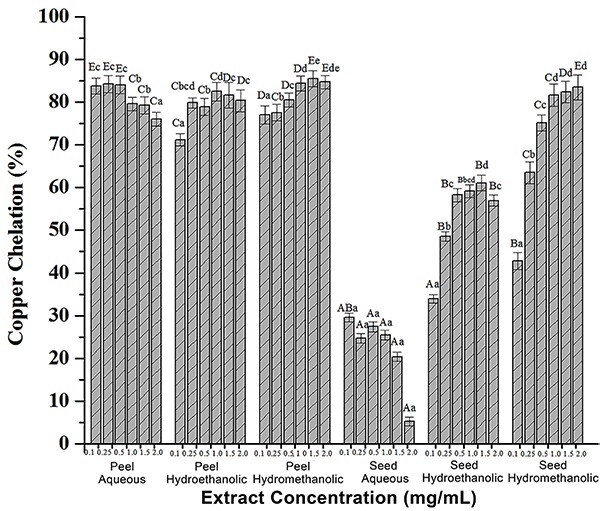
Antioxidant activity of melon peel and seed extracts by copper chelating effect. Different lower-case letters indicate a significant difference between melon residues extracts when compared at each concentration. Different upper-case letters indicate a significant difference between melon residues extracts at the same concentration (P<0.05, ANOVA). Data are reported as means±SD of 3 determinations.

All peel extracts showed copper-chelating activity above 50%. The aqueous extract showed a non-dose-dependent ability, with increased activity at a 0.25 mg/mL concentration (84.25%). For hydroethanolic and hydromethanolic extracts, the highest activities were found at 1.0 and 1.5 mg/mL concentrations (Figure 4). For seed extracts, the hydromethanolic showed chelating activity of copper ions from the 0.25 mg/mL concentration, with a dose-dependent action, reaching chelation values of 83.43% at the highest concentration (2.0 mg/mL). In contrast, the seed aqueous extract did not show satisfactory ability to chelate copper, with maximum activity of 29.5% in the 0.1 mg/mL concentration. These findings are important to improve exogenous antioxidants, mainly obtained from diet, to inhibit or reduce the effects of reactive oxygen species (36).

Melon peel methanolic and ethanolic extracts showed hydroxyl radical sequestering activity in dose-dependent manner. The ethanol extract showed high sequestering activity with 50.56% at the 1.0 mg/mL concentration and 67.69% at 2.0 mg/mL. However, the methanol extract had a low activity of 5.09 and 7.04% at 0.5 and 1.5 mg/mL, respectively. Similarly, the aqueous extract showed low activity, and only the 0.5 mg/mL concentration presented a sequestering ability of the hydroxyl radical of 3.52%. None of the extracts from melon seed flour presented hydroxyl radical elimination activity. Gallic acid showed 28% sequestration effect of the hydroxyl radical at 0.5 mg/mL and 95% at 1.0 mg/mL (data not shown).

Table 2 shows a high antioxidant capacity for hydromethanolic and ethanolic extracts of melon peel in the DPPH test, a relatively stable radical due to displacement of a free electron in its entire molecule, with no dimerization as occurs with most free radicals. The decrease in absorbance of DPPH in the presence of fruit waste in a 30-min reaction was also verified by Mosmann (18) who found DPPH sequestration ability for lychee seed (80%), grape seed (77%), lychee peel (71%), banana peel (43%), and BHT (83%). Regarding the reactive oxygen antioxidant capacity and the antioxidant scavenging activity against the peroxyl radical induced by 2,2′-azobis-2-amidinopropane dihydrochloric at 37°C assessed by a fluorescent test with fluorescein, the loss of fluorescence is an indicator of the extent of peroxyl radical decomposition. The free radical scavenging/oxygen radical absorbance capacity of a compound may serve as a significant indicator of its potential antioxidant activity (37). In this study, we found that the extracts possessed free radical scavenging capabilities; however, the seed aqueous extract exhibited low fluorescein decay (Table 2).

### Antiproliferative activity

Numerous studies have demonstrated the relationship between antioxidant and antiproliferative activities of plant compounds (34). Strategies for chemoprevention of cancer using natural foods, mainly plant residues, are regarded as one of the most important fields for cancer control (3). To evaluate the antiproliferative potential of melon seed and peel extracts, the following human tumor cell lines were chosen: SiHa, HeLa, HT-29 and 786–0. The inhibition of cell proliferation occurred in the presence of various concentrations of the extracts assessed by MTT assay. This system, based on the reduction of the tetrazolium derivatives in living cells by mitochondrial dehydrogenases, allows the estimation of the activity of metabolically active cells. This assay measures cell viability and survival (cell counting), and cells proliferation (cell culture assays), indicating mammalian cell survival and growth.

**Figure 5.**
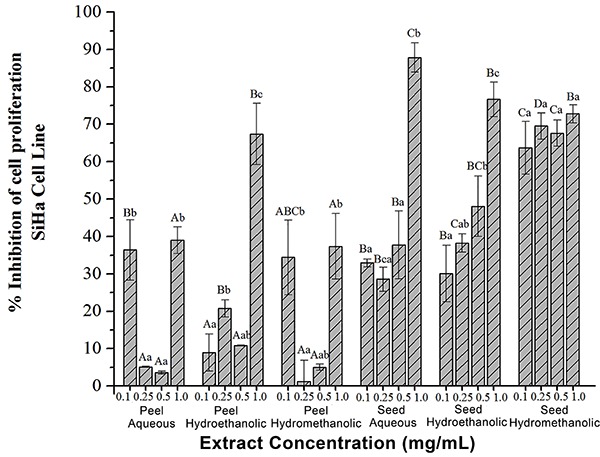
Effect of extracts of melon residues on the inhibition of cell proliferation of the SiHa cell line, measured by MTT test. Cells proliferation was carried out in the presence of melon residues extracts in different concentrations for 24 h. ^a,b,c,d^Significant differences between different concentrations of the same sample. ^A,B,C,D^Significant differences between different extracts in the same concentration (P<0.05, ANOVA). Data are reported as means±SD.

To evaluate the toxicity of compounds against normal cells, we used the cell line 3T3. The data showed that the melon peel and seed extracts had low toxicity in those cells. Inhibition of the proliferation reached a maximum range of 25% at 1.0 mg/mL in 24 h (data not shown). SiHa cells inhibition was observed for all seed extracts at a 1.0 mg/mL concentration with activity above 80% in aqueous extracts, 75% for the hydroethanolic extract, and 72% for hydromethanolic. In contrast, the extracts of melon peel had cell inhibition activities detected only for the hydroethanolic extract in the 1.0 mg/mL concentration with 65% inhibition. Inhibition of tumor cell proliferation occurred in the presence of various concentrations of the extracts. The peel hydroethanolic extract and seed aqueous extract presented cell inhibition activity in a dose-dependent manner for cervical tumor cell lines SiHa (Figure 5). IC_50_ values of 1.2 and 0.4 mg/mL were noted for peel hydroethanolic extract and seed hydromethanolic extract, respectively.

For SiHa cell line, the peel aqueous extract showed slight inhibition of cell proliferation at all concentrations by at least 50%. Melon peel hydroethanolic extracts were significantly different from other extracts showing higher antiproliferative activity against SiHa cervical tumor cells in high concentrations (0.5 to 1.0 mg/mL) with inhibition percentage of around 55–60%, respectively (P<0.05). Results for seed extracts were statistically higher than for peel extracts. Melon seed extract was more active against tumor cells, showing cellular inhibition above 70% for the methanol solution, and significant data at the of 0.5 and 1.0 mg/mL concentrations of (P<0.05).

**Figure 6.**
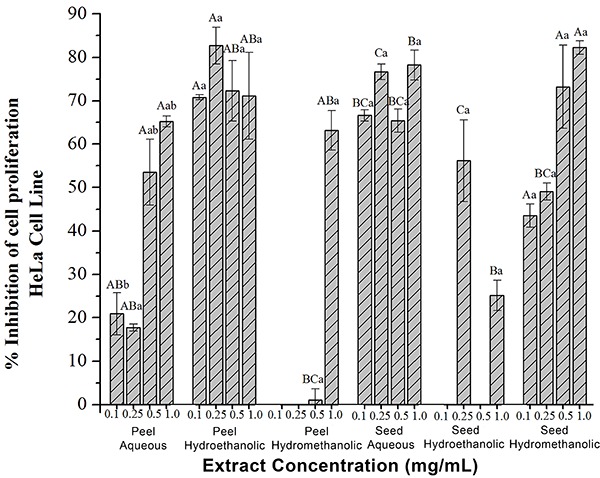
Effect of melon residues extracts on the inhibition of cell proliferation of the HeLa cell line, measured by MTT test. Cells proliferation was carry out in the presence of melon residues extracts in different concentrations for 24 h. ^a,b,c,d^Significant differences between different concentrations of the same sample. ^A,B,C,D^Significant differences between different extracts in the same concentration (P<0.05, ANOVA). Data are reported as means±SD.

Regarding cytotoxicity assays for human cervical adenocarcinoma HeLa cells, the peel aqueous extract showed proliferation inhibition activity greater than 50% at 0.5 and 1.0 mg/mL concentrations. The peel hydroethanolic extract showed activity in all concentrations, particularly at the 0.25 mg/mL concentration, with capacity above 80%. However, the methanolic extract showed an activity greater than 50% in proliferation inhibition only in the 1.0 mg/mL concentration (Figure 6).

The seed hydromethanolic extract was a highlight, as it reached more than 80% cell proliferation inhibition of HeLa cells dose-dependently until a concentration of 1.0 mg/mL. The aqueous extract followed with 80% inhibition. The seed aqueous extract showed significant difference for inhibition at all concentrations, reducing cell proliferation by 60% (P<0.05). The hydromethanolic extract showed dose-dependent activity against HeLa cells, with different inhibition percentages above 70% at 0.5 and 1.0 mg/mL concentrations. For HeLa cells, the IC_50_ values were lower for melon peel extracts (0.5 mg/mL) than seed extracts (0.3 mg/mL).

Antitumor biological activity exerted by melon residues can be explained by the presence of phenolic content, such as flavonoids and tannins, and the high antioxidant capacity demonstrated by *in vitro* assays. Cases involving high levels of oxidative stress or DNA damage have been reported in human malignancies. A study investigating cervical cancer experimental model and cervical tissue cells from human patients found that the levels of 8-oxodG increased significantly (P<0.001) in low-grade DNA or high-grade dysplasia in tumors compared to normal. The 8-oxo compound 2′deoxiguanosina (8oxodG) is a cancer molecular marker induced when there is damage to DNA and acts as oxidative stress flag, as it is the main DNA oxidation product (38).

**Figure 7.**
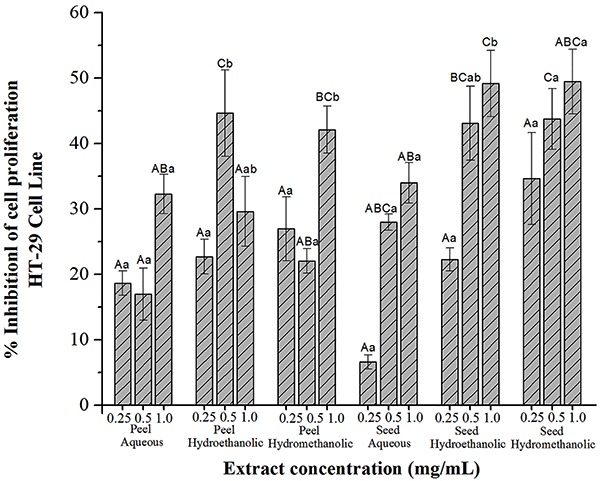
Effect of melon residues extracts on the inhibition of cell proliferation of the HT-29 cell line, measured by MTT test. Cells proliferation was carry out in the presence of melon residues extracts in different concentrations for 24 h. ^a,b,c,d^Significant differences between different concentrations of the same sample. ^A,B,C,D^Significant differences between different extracts in the same concentration (P<0.05, ANOVA). Data are reported as means±SD.

Concerning colon tumor growth type HT-29 cells, all extracts and all concentrations tested exhibited activity below 50%. Thus, for this type of cell, higher concentrations of the extracts must be investigated, aiming at a possible activity, since the 0.25, 0.5, and 1.0 mg/mL concentrations showed dose-dependent responses (Figure 7). For this cell line, it is estimated that IC_50_ values should be 4.0 and 2.8 mg/mL for peel and seed extracts, respectively.

**Figure 8.**
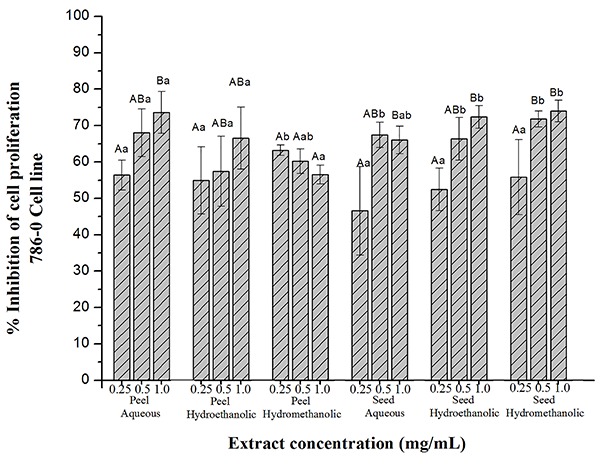
Effect of melon residues extracts on the inhibition of cell proliferation of the 786–0 cell line, measured by MTT test. Cells proliferation was carry out in the presence of melon residues extracts in different concentrations for 24 h. ^a,b,c,d^Significant differences between different concentrations of the same sample. ^A,B,C,D^Significant differences between different extracts in the same concentration (P<0.05, ANOVA). Data are reported as means±SD.

For tests with human renal tumor type 786–0 after 24 h, with the exception of the aqueous seed extract at the lowest concentration, all extracts showed inhibition of cell growth greater than 50%, particularly with the 1.0 mg/mL concentration, which had inhibition rates ranging from 50–80%. In this assay, the IC_50_ value for seed extracts was 1.0 mg/mL and for peel extract was 0.4 mg/mL. However, there was no statistical difference between the different studied extracts (P<0.05; Figure 8).

Antiproliferative activity is possibly related to the amount of phenolic compounds present in the extracts. Phenolic compounds from plant extracts have recently been appointed as antiproliferative agents. A high correlation between the quantities of phenolic compounds present in extracts and antitumor analysis was found by Chon et al. (39). In this study, we observed antiproliferative effects for melon residues extracts on tumor cells that might have been caused by the activation of cell death mechanisms.

Several antioxidant mechanisms from compounds present in melon peels and seeds might be associated with antiproliferative effects. Several enzymes, such as cytochrome C oxidase, ascorbate oxidase and superoxide dismutase, are involved in cellular redox mechanisms. Therefore, copper is used as the electron transporter in biological systems. Patients with breast, cervical, ovarian, lung, prostate, and stomach cancers, and leukemia showed high copper concentrations in both serum and tissues (40). An effective copper chelation is the most appropriate and effective treatment of carcinogenicity induced by copper, being effective also in neurodegenerative and chronic diseases.

The generation of ROS via Fenton reaction is the most important mechanism of toxicity mediated by iron. The Fe^2+^ present in the cell catalyzes the production of hydroxyl radicals via the Fenton reaction, based on the hydrogen peroxide production mainly by the dismutation of superoxide dismutase. Inefficient iron chelation can result in the formation of toxic free radicals. A recent research has sought to develop new iron chelators, in which the di-2-piridilcetone thiosemicarbazone and 2-benzoilpiridina were found as potential chelating agents for use in cancer treatments (41).

The results of this study indicated that melon peels and seeds extracted in different solvents have several activities against different tumor cell lines, showing that the compounds could be promising antitumor agents. Furthermore, melon residues might be an important dietary element for health promotion in general and possible prevention of diseases such as cancer.

In conclusion, we have shown that residues of *Cucumis melo* L. var. *reticulatus* exhibited properties that can promote antioxidative reactions and anticancer effects. The presence of phenolic compounds possibly explains the antioxidant potential found in both melon peel and seed extracts. In addition, their bioactive content may also support the antiproliferative effects in the tumor cell lines studied.

The results of this study contribute to the area of functional foods, since it provided findings for a new potential use for melon residues, especially its role in cancer prevention. Moreover, melon peel and seed extracts showed good metal chelating ability that can be interpreted as an antioxidative feature. The very promising results regarding the *in vitro* antioxidative activity are fundamental to the application in the food industry, as well as in herbal medicines. Melon residues extracts possess a high content of total phenolic compounds. Peel and seed extracts using different solvents displayed antioxidant activity and can be applied in food technology, pharmacology and medicine studies. Besides having good technological properties for the development of new products aimed at processing food waste, extracts of melon residues exhibited antiproliferative capacity against tumor cells such as human kidney, colon and cervical cancers.
